# Supplementary material for: Only Words Count; the Rest Is Mere Chattering: A Cross-Disciplinary Approach to the Verbal Expression of Emotional Experience
Source: Behav Sci (Basel). 2022 Aug 18;12(8):292. doi: 10.3390/bs12080292 (PMC9404916; doi:10.3390/bs12080292)
Supplement: Supplementary file 1 [file behavsci-12-00292-s001.zip › Supplementary Figure S1_S2_S3_S4 Supplementary Table S1_S2B.pdf]

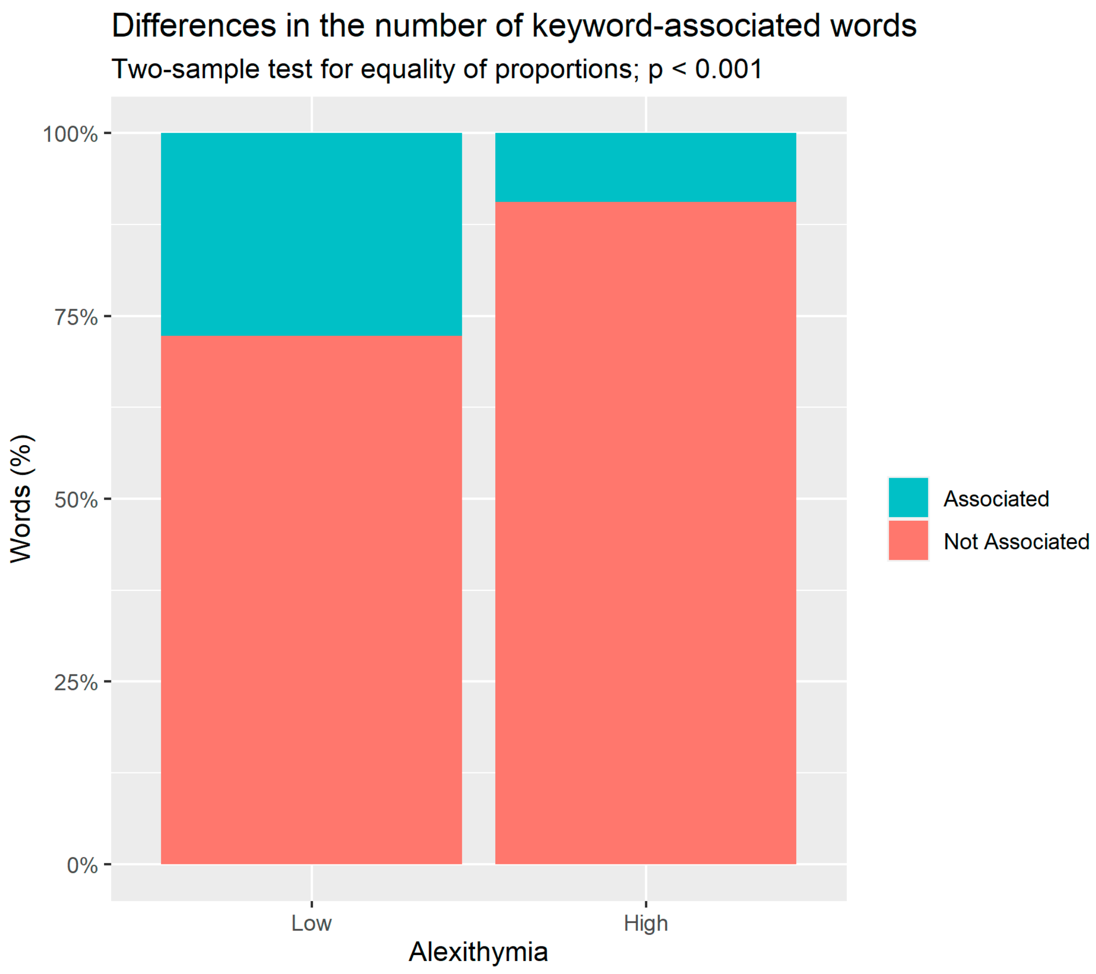

Supplementary Figure S1. The relation between alexithymia and keywords related to emotional expression was detailed by Target Word Collocation Analysis. The resulting network was smaller in subjects with high alexithymia levels ($p<0.001$) than in subjects with low alexithymia levels.

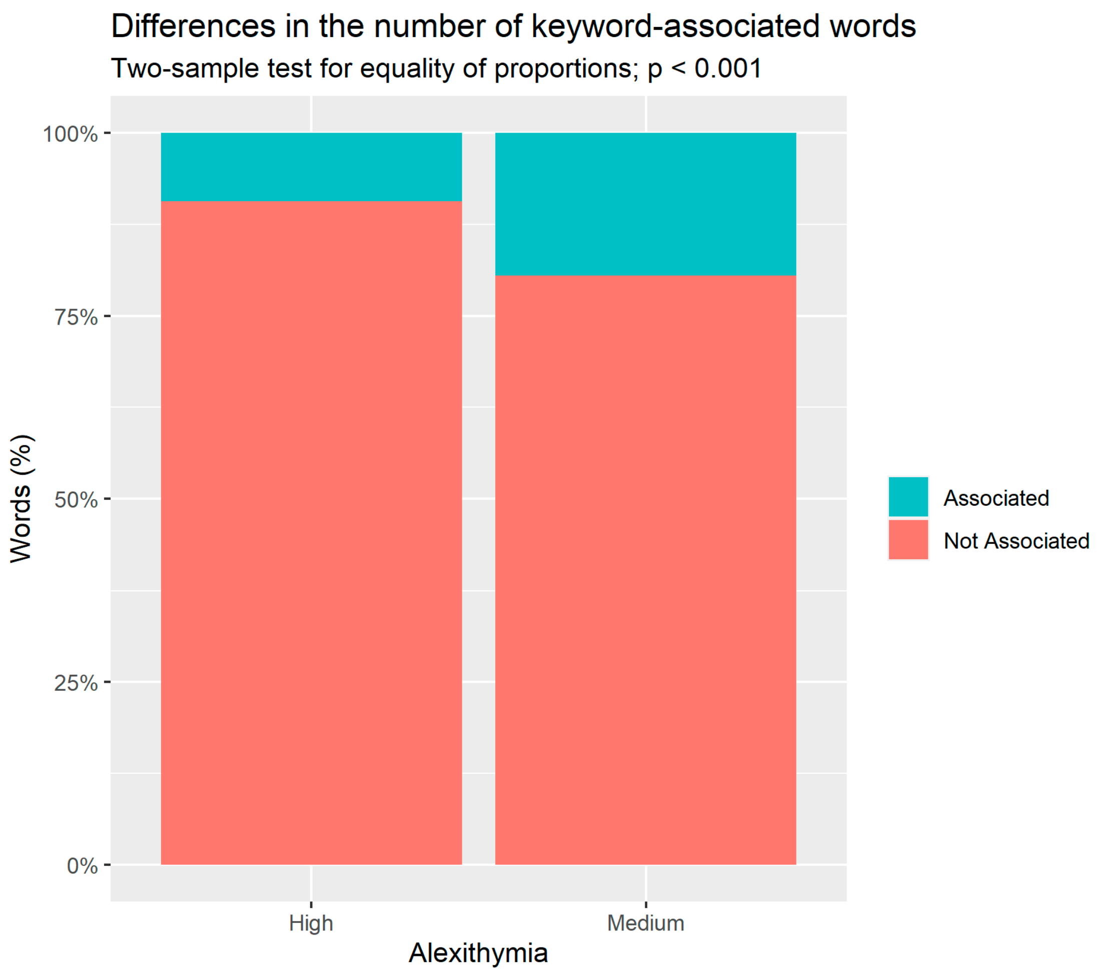

Supplementary Figure S2. The relation between alexithymia and keywords related to emotional expression was detailed by Target Word Collocation Analysis. The resulting network was smaller in subjects with high alexithymia levels ($p<0.001$) than in subjects with medium alexithymia levels.

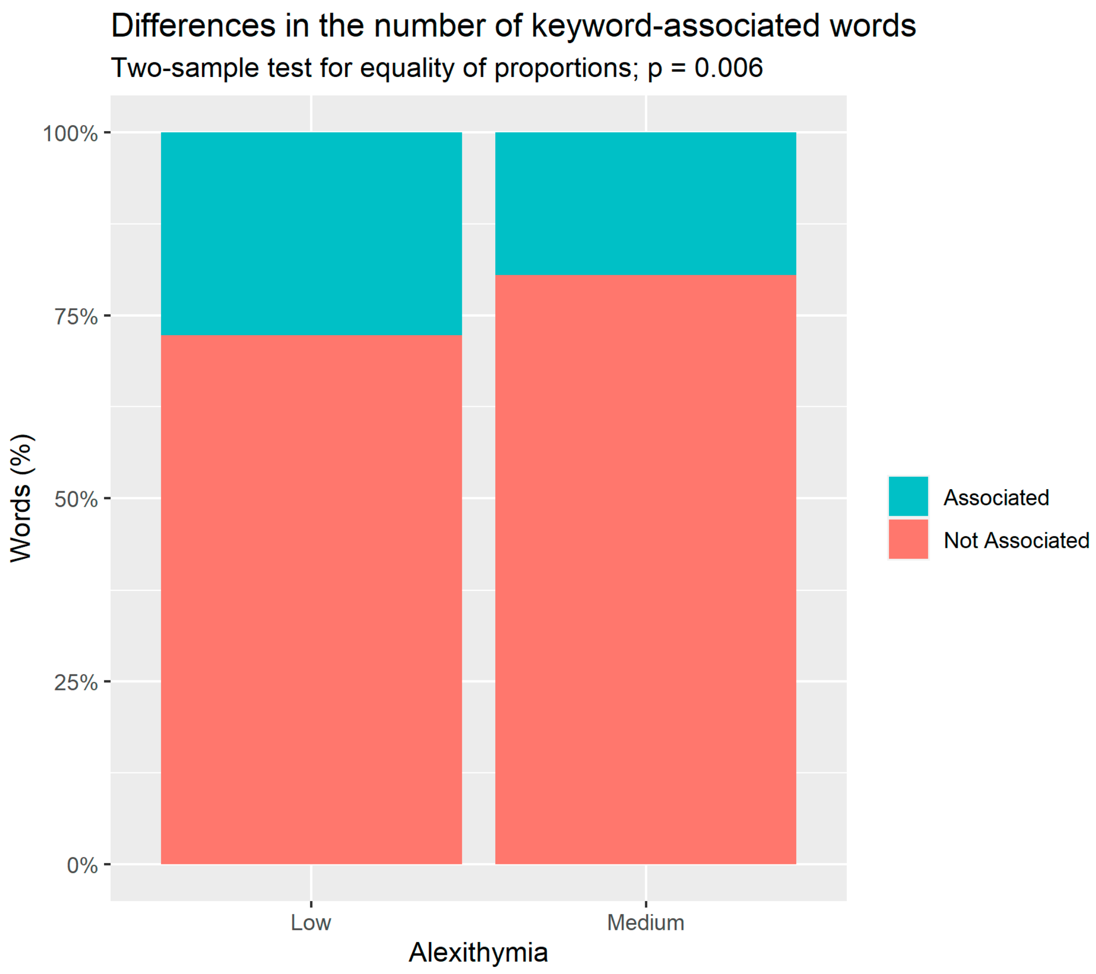

Supplementary Figure S3. The relation between alexithymia and keywords related to emotional expression was detailed by Target Word Collocation Analysis. The resulting network was smaller in subjects with medium alexithymia levels (p=0.006) than in subjects with low alexithymia levels.

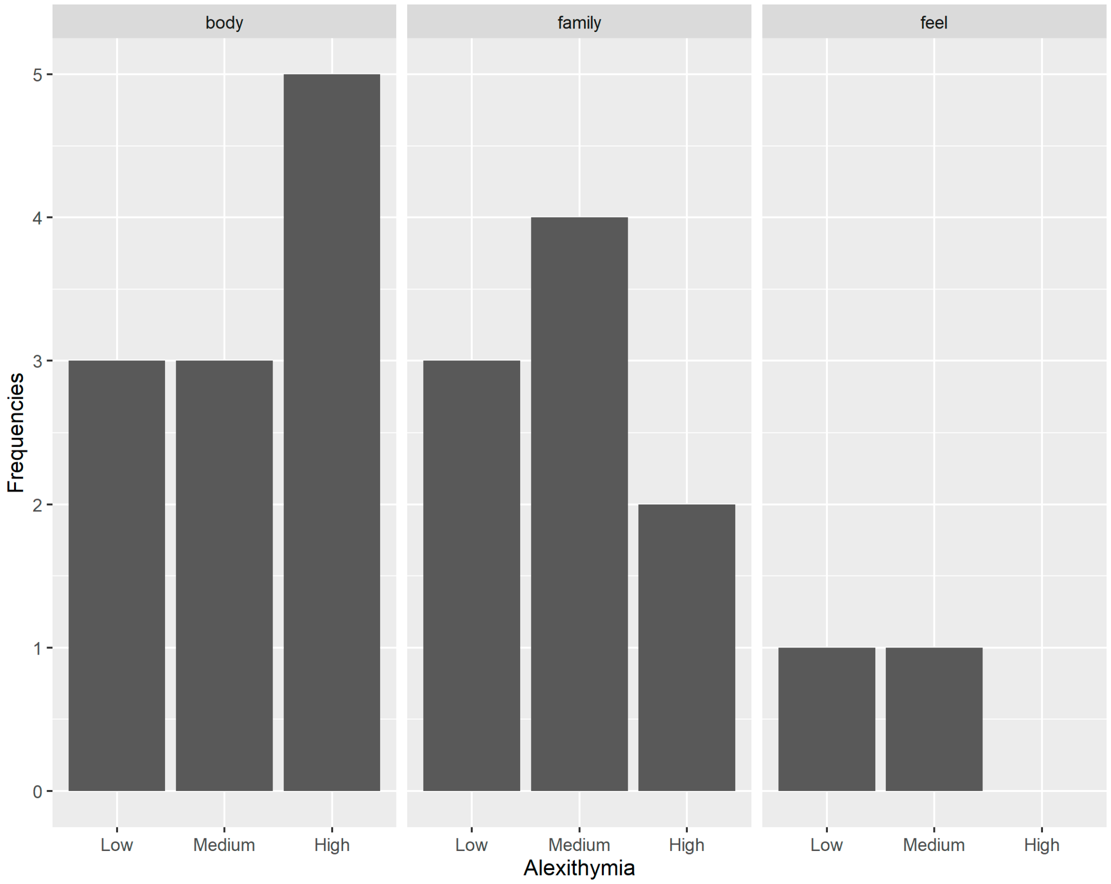

Supplementary Figure S4. Seeded Latent Dirichlet Allocation, we built three dictionaries containing words related to bodily parts or sensations (“body”), relational words (“family”) or emotional words (“feel”). Subjects with high alexithymia levels showed elevated frequencies of “body”-related words and no “feeling”-related words.

		Total Sample	High Alexithymia	Medium Alexithymia	Low Alexithymia
	**Number** *(Males)*	22 (12)	7 (6)	8 (4)	7 (2)
	**Age** *Mean in years (±SD)*	44.32 (13.17)	52.14 (4.98)	41.88 (12.90)	39.29 (16.70)
	**Years of formal education** *Mean in year (±SD)*	15.68 (2.40)	15.00 (1.73)	15.13 (3.23)	17.00 (1.41)
TSIA *Mean (±SD)*	**Total Score**	26.41 (13.86)	41.71 (2.14)	27.88 (6.31)	9.43 (2.88)
	**Difficulty Identify Feelings (DIF)**	5.36 (3.71)	9.43 (1.27)	5.75 (1.39)	0.86 (1.07)
	**Difficulty Describing Feelings (DDF)**	7.18 (4.66)	11.71 (0.49)	8.38 (2.26)	1.29 (1.80)
	**External Oriented Thinking (EOT)**	6.32 (4.29)	10.57 (1.13)	7.13 (2.59)	1.14 (1.46)
	**Imaginative Process (IP)**	7.55 (3.00)	10.00 (1.41)	6.63 (2.45)	6.14 (3.48)
ASQ *Mean (±SD)*	**Trust**	34.27 (6.57)	31.86 (9.49)	32.50 (3.59)	38.71 (3.45)
	**Discomfort with Intimacy**	32.52 (6.29)	36.00 (5.88)	32.94 (6.25)	28.57 (5.06)
	**Relationships as Secondary**	14.80 (4.36)	15.57 (4.23)	17.06 (2.65)	11.43 (4.43)
	**Need for Approval**	20.23 (5.14)	20.43 (2.23)	22.13 (5.46)	17.86 (6.41)
	**Preoccupation with Relationships**	28.05 (5.36)	28.14 (3.13)	29.63 (5.90)	26.14 (6.52)
ERQ *Mean (±SD)*	**Cognitive Reappraisal**	30.61 (4.90)	31.43 (2.05)	28.19 (6.05)	32.57 (4.89)
	**Expressive Suppression**	12.61 (4.73)	15.00 (3.86)	13.31 (5.42)	9.43 (3.15)
COPE *Mean (±SD)*	**Problem Solving**	34.77 (3.10)	36.43 (2.30)	33.50 (3.82)	34.57 (2.44)
	**Turning to Religion**	21.09 (5.96)	21.57 (7.57)	21.38 (5.42)	20.29 (5.62)
	**Positive Attitude**	33.36 (2.56)	33.43 (2.88)	33.63 (2.33)	33.00 (2.83)
	**Seeking Social Support**	35.52 (7.22)	36.29 (8.82)	33.44 (6.74)	37.14 (6.49)
	**Avoidance**	26.32 (4.67)	27.57 (5.32)	27.88 (4.12)	23.29 (3.55)
	**BDI** *Mean (±SD)*	3.64 (3.37)	2.71 (2.29)	4.25 (3.49)	3.86 (4.34)
	**STAI-2** *Mean (±SD)*	34.05 (7.99)	29.00 (8.10)	35.75 (5.44)	37.14 (8.86)

Supplementary Table S1. Demographic variables (gender, age, and years of formal education) and scores of psychological scales [Toronto Structured Interview for Alexithymia – TSIA (total and factors: Difficulty in Identifying Feelings - DIF; Difficulty in Describing Feelings - DDF; Externally Oriented Thinking - EOT; and Imaginal Processes – IP); Attachment Style Questionnaire - ASQ, Emotion Regulation Questionnaire - ERQ, Coping Orientation to Problems Experiences - COPE, Beck Depression Inventory - BDI, and State-Trait Anxiety Inventory-2 - STAI-2] of the participants (total sample and divided in three groups based on their overall TSIA scores: subjects with total TSIA scores ≤13: Low Alexithymia subjects; subjects with total TSIA score >13 and <39: Medium Alexithymia subjects; subjects with total TSIA score ≥39: High Alexithymia subjects).

**Energy related LLDs (4)**	**Group**
Sum of auditory spectrum (loudness)	Prosodic
Sum of RASTA-filtered auditory spectrum	Prosodic
RMS Energy	Prosodic
**Spectral LLDs**	**Group**
Zero-Crossing Rate	Prosodic
RASTA-style auditory spectrum, bands 1-26 (0-8 kHz)	Spectral
Spectral energy 250–650 Hz, 1 k–4 kHz	Spectral
Spectral roll-off point 0.25, 0.5, 0.75, 0.9	Spectral
Spectral Flux, Centroid, Entropy, Slope	Spectral
Psychoacoustic Sharpness, Harmonicity	Spectral
Spectral Variance, Skewness, Kurtosis	Spectral
MFCC 1–14	Cepstral
**Voicing related LLDs**	**Group**
F_0 (SHS & Viterbi smoothing)	Prosodic
Prob. of voicing	Sound quality
log. HNR, Jitter (local, delta), Shimmer (local)	Sound quality

Supplementary Table S2B. The ComParE acoustic feature set provides 65 low-level descriptors (LLDs) divided into 4 groups: prosodic, spectral, cepstral and voice quality.
