# Supplementary figures and images for: Only Words Count; the Rest Is Mere Chattering: A Cross-Disciplinary Approach to the Verbal Expression of Emotional Experience
Source: Behav Sci (Basel). 2022 Aug 18;12(8):292. doi: 10.3390/bs12080292 (PMC9404916; doi:10.3390/bs12080292)

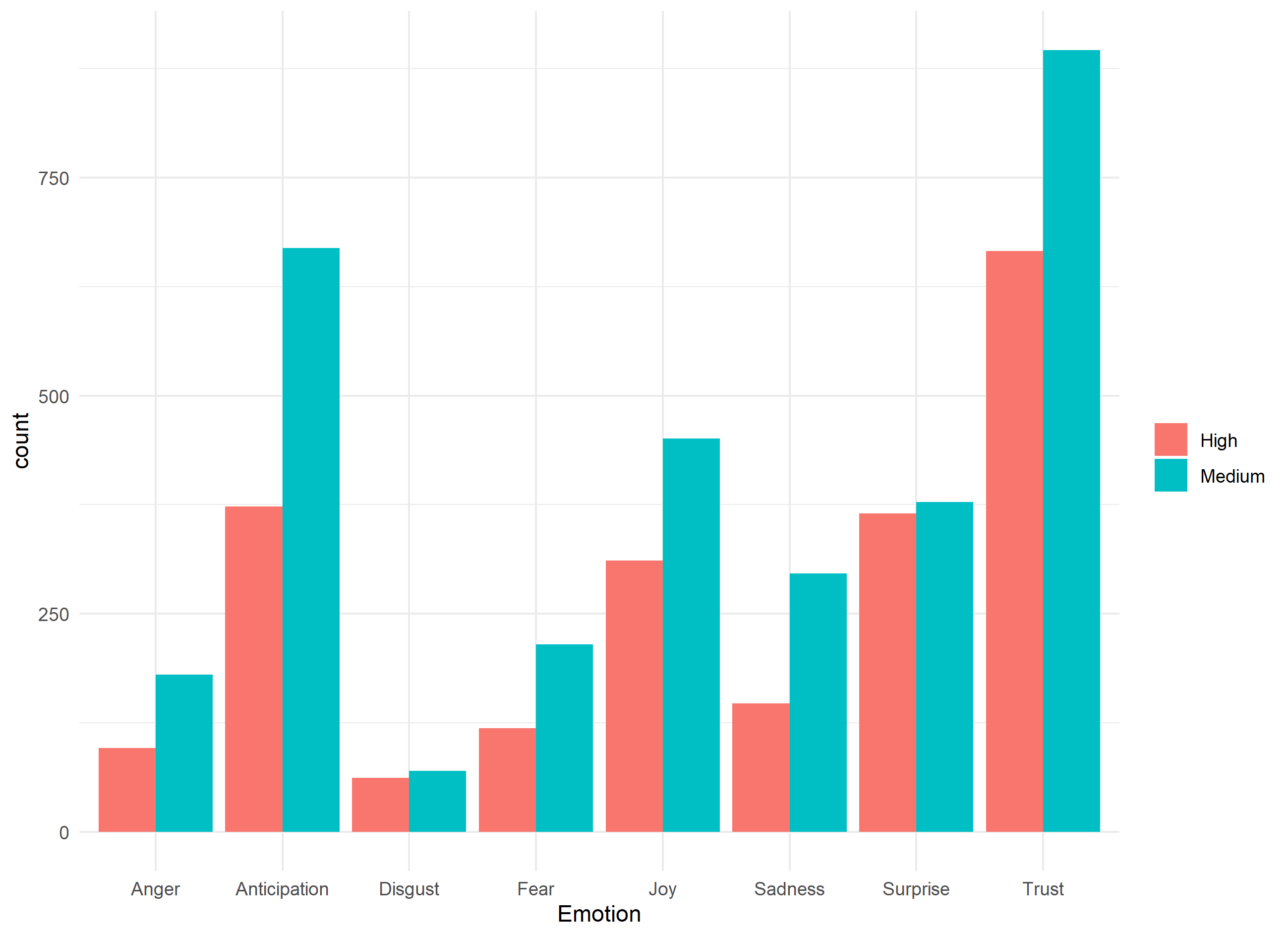

Supplement: Supplementary file 1 [file behavsci-12-00292-s001.zip › SupplRes5 Emotion Recognition HA vs MA.png]

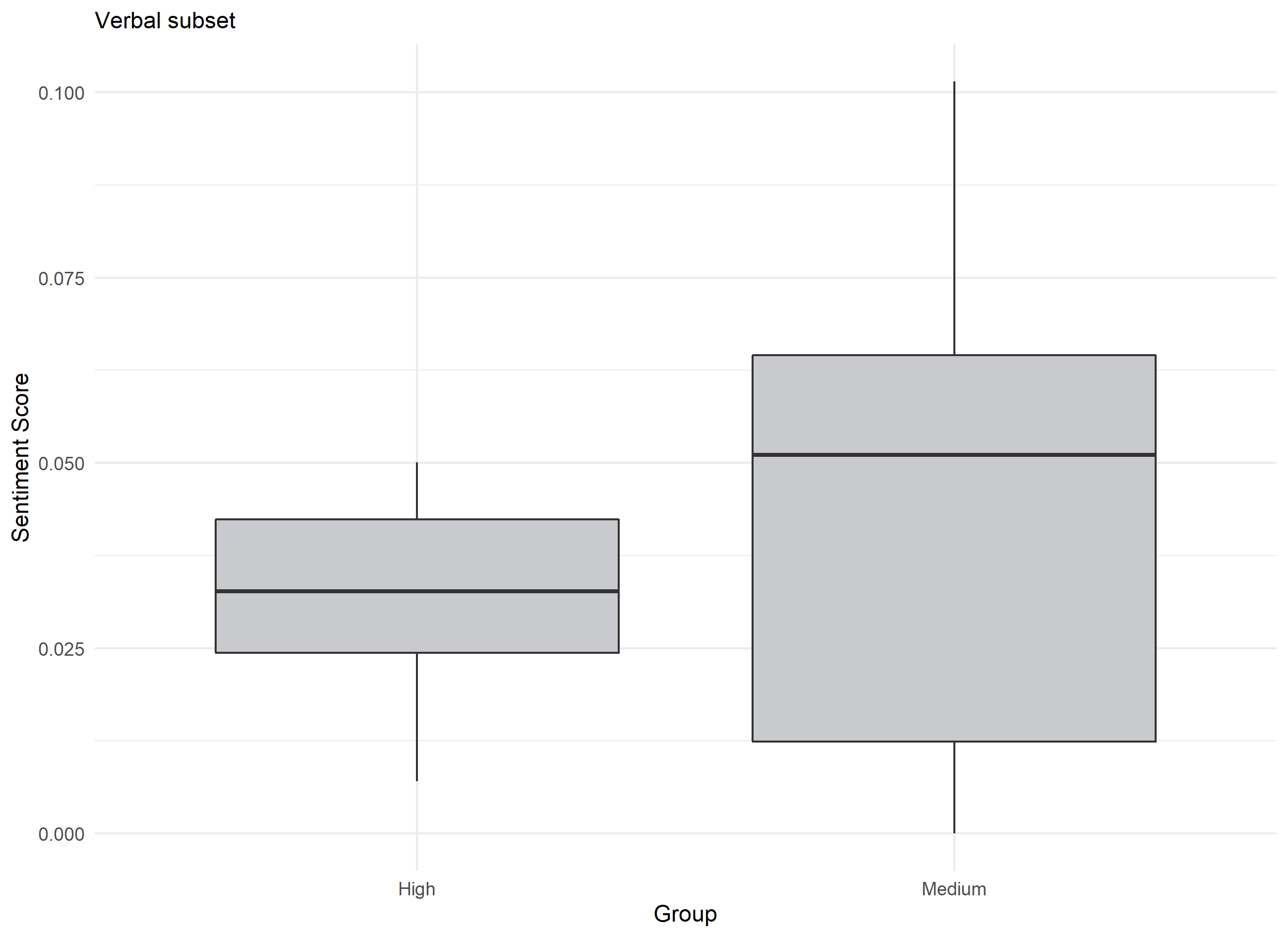

Supplement: Supplementary file 1 [file behavsci-12-00292-s001.zip › SupplRes5 Sentiment Analysis HA vs MA.png]

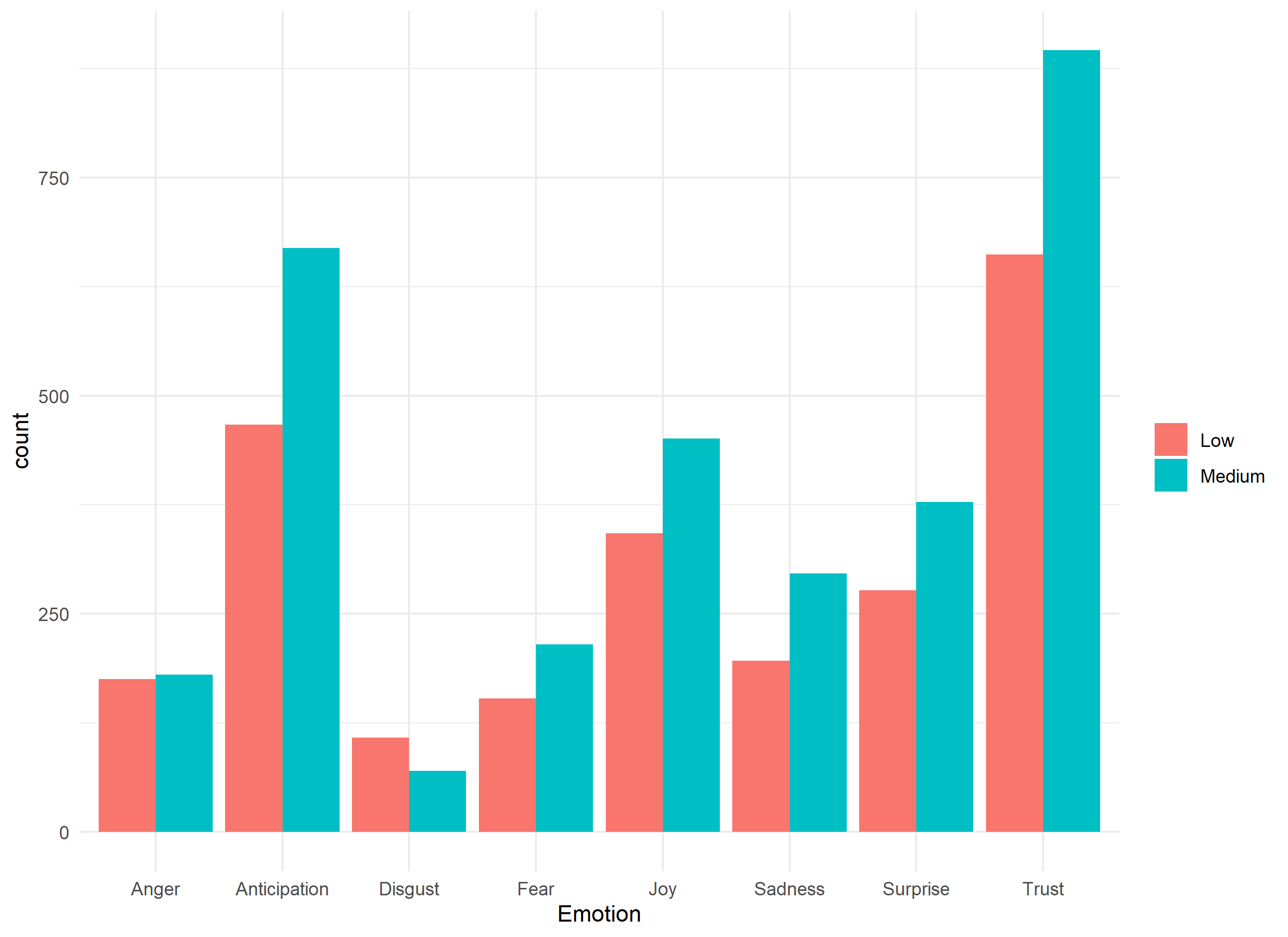

Supplement: Supplementary file 1 [file behavsci-12-00292-s001.zip › SupplRes6 Emotion Recognition LA vs MA.png]

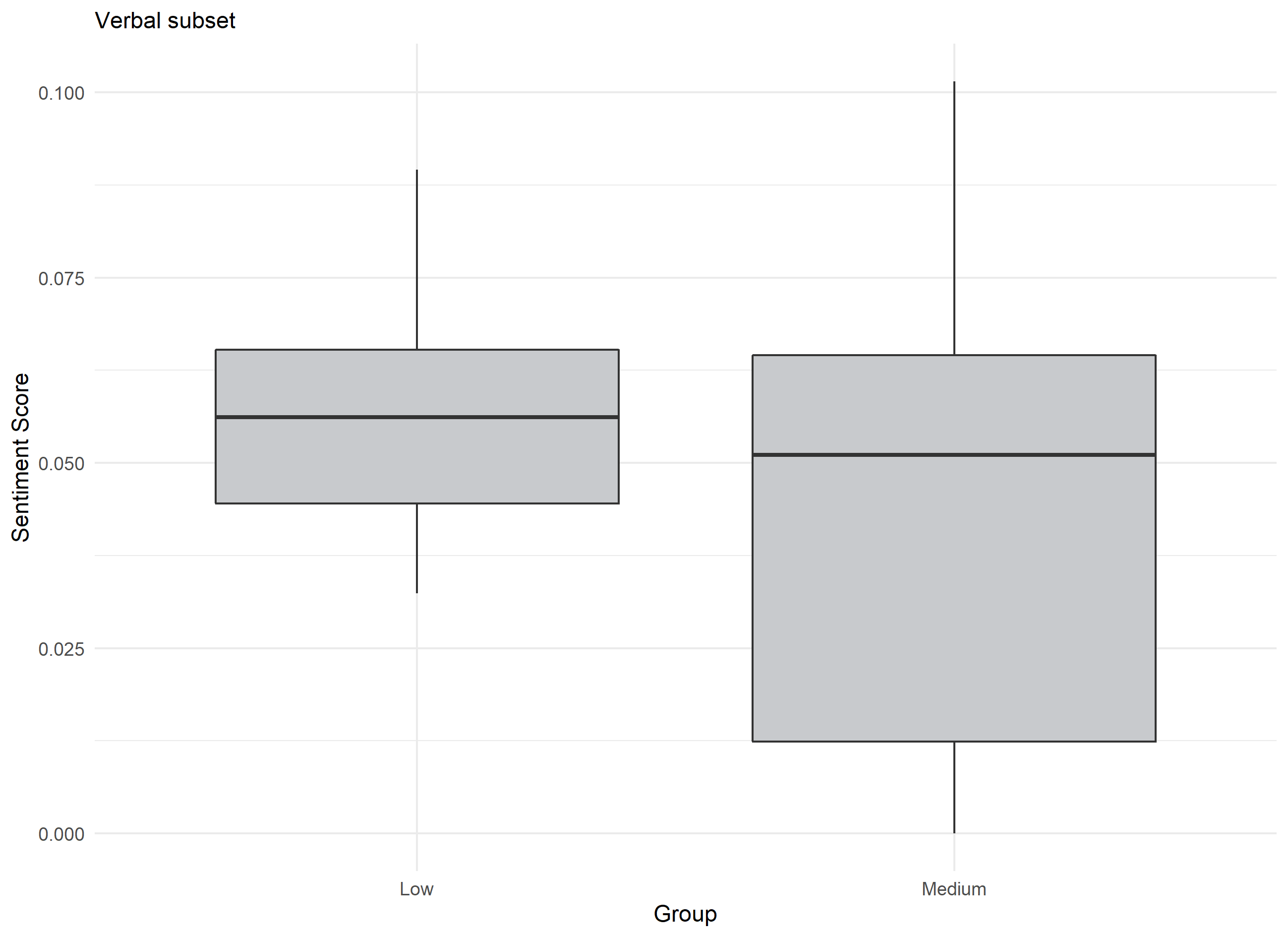

Supplement: Supplementary file 1 [file behavsci-12-00292-s001.zip › SupplRes6 Sentiment Analysis LA vs MA.png]
